# Micro-PET Imaging Demonstrates 3-*O*-β-D-Glucopyranosyl Platycodigenin as an Effective Metabolite Affects Permeability of Cell Membrane and Improves Dosimetry of [^18^F]-Phillygenin in Lung Tissue

**DOI:** 10.3389/fphar.2019.01020

**Published:** 2019-09-13

**Authors:** Fukui Shen, Wenbo Wu, Man Zhang, Xiaoyao Ma, Qingxin Cui, Zhongyao Tang, Hao Huang, Tiantian Tong, Leefong Yau, Zhihong Jiang, Yuanyuan Hou, Gang Bai

**Affiliations:** ^1^State Key Laboratory of Medicinal Chemical Biology, College of Pharmacy and Tianjin Key Laboratory of Molecular Drug Research, Nankai University, Tianjin, China; ^2^State Key Laboratory of Quality Research in Chinese Medicine, Macau University of Science and Technology, Macau, China

**Keywords:** *Platycodon grandiflorum*, secondary saponin, 3-*O*-β-D-glucopyranosyl platycodigenin, [^18^F]-phillygenin, micro-positron emission tomography

## Abstract

*Platycodon grandiflorum*, as a traditional medicinal plant, is commonly used in the treatment of pulmonary disease. Platycodon saponins are proposed as active ingredients. However, the role of secondary saponin metabolites (SSM) in the traditional use of *Platycodon* has not yet been fully clarified. In this study, [^18^F]-phillygenin ([^18^F]-PH) probe was synthesized and thereby used as a tracer for micro-positron emission tomography scanning to explore the effects of platycodon saponins. The membrane permeability with different SSM was evaluated *in vitro* based on the dye-carrying capacity of fluorescein isothiocyanate. The results showed that total platycodon saponins improved the dosimetry of [^18^F]-PH in the lung tissue, and an SSM named 3-*O*-β-D-glucopyranosyl platycodigenin (GPD^682^) appreciably changed the distribution of drugs both *in vitro* and *in vivo*. We propose that GPD^682^ could be utilized as an important ingredient to help drug delivery to the lung tissue and improve the treatment of respiratory disease.

## Introduction

Traditional Chinese medicine (TCM) formulas are the primary clinical mode of TCM, and many Chinese herbal formulas have good clinical effects (Chen et al., 2019). The core of the formula is prescription compatibility, which is the compatibility of medicine pairs (Wang et al., 2012). According to this theory, the interactions between different herbs have synergistic effects, can reduce toxicity, and increase efficiency (Liu et al., 2016; Huang et al., 2019). For example, long-term use of *Radix Glycyrrhizae* (Gancao) alone can cause adverse reactions such as electrolyte metabolism disorder (Harding and Stebbing, 2017). However, in the Gancao Kushen decoction, a TCM formula for the treatment of hepatitis and liver fibrosis, *Radix Sophorae flavescentis* (Kushen) plays a vital role in slowing absorption and accelerating the metabolism of Gancao main activity ingredients (Shi et al., 2015). Jiegeng Gancao decoction comprises *Radix Platycodonis* (Jiegeng) and *Radix Glycyrrhizae* (Gancao) at a weight ratio of 1:2 and is widely used to treat sore throat and cough (Yang et al., 2017). Jiegeng affects the pharmacokinetic properties of flavonoids and saponins in Gancao and alters the curative effect (Mao et al., 2017).

*Radix Platycodonis* is the dry root of *Platycodon grandiflorum* (Jacq.) A. DC., which is widely grown in China, Japan, and Eastern Siberia. *P. grandiflorum* (PG) as a medicine food homology has been widely accepted and utilized (Lee et al., 2004; Zhang et al., 2015; Liu, 2018). It has a long medicinal history and is often used as an adjunct in treatment of respiratory disease (Yim et al., 2016), and a recent study revealed that PG could affect the distribution of glycyrrhetinic acid in organisms (Cui et al., 2018). Pharmacokinetic studies have shown that saponins in PG are quickly metabolized into secondary saponin metabolites (SSM) in the body and then produce pharmacological effects (Tang et al., 2017), and it has been indicated that SSM might be the main medicinal substance (Lorent et al., 2014). Although dozens of active SSM have been reported in PG, few previous studies have revealed the specific compounds that exert the activity.

Strategies based on liquid chromatography-mass spectrometry (LC-MS) (Cunha et al., 2014) and proton nuclear magnetic resonance (^1^H-NMR) (Lei et al., 2015; Hirakawa et al., 2019) are often time-consuming and require a large number of animal experiments, as they are designed to test metabolites in blood after *in vivo* metabolism. Besides, the vast differences in animal individuals increased the difficulty of dynamic observation (Cunha et al., 2014). Micro-positron emission tomography (Micro-PET) studies the pharmacokinetics in animals by capturing radiolabeled nuclides-tagged imaging molecules (Nabulsi et al., 2019). Now Micro-PET is a particularly useful functional imaging tool with the advantages of high sensitivity, no trauma, and quantitative dynamic observation (Zullo et al., 2018) and is therefore widely used for *in vivo* study (Yang et al., 2019; Zhang et al., 2019).

In TCM formulas, PG is often compatible with *Forsythia fructus* (Lianqiao) for the treatment of respiratory disease (Li et al., 2013). The main active component phillyrin of *F. fructus* can be hydrolyzed into phillygenin (PH) *in vivo* (Lee et al., 2011), which is regarded as the fundamental pharmacological basis targeting AKT for anti-inflammatory therapy (Liu et al., 2017). In order to explain the synergistic mechanism and find out whether PG can improve the pharmacokinetic distribution of PH and play an auxiliary role, in this study, an [^18^F]-phillygenin ([^18^F]-PH) probe was synthesized and used as a tracer for Micro-PET scanning to explore the effects of platycodins. After oral administration of PG, the main metabolic derivative SSM was separated, and membrane permeability assay *in vitro* was performed at the cell level. Finally, the activity of the monomeric compound was verified by Micro-PET. The results showed that a key SSM, 3-*O*-β-D-glucopyranosyl platycodigenin (GPD^682^), can appreciably improve the distribution of drugs both *in vitro* and *in vivo*.

## Materials and Methods

### Chemicals and Reagents

Phillygenin (PH) was purchased from Pfeide Biotechnology Co., Ltd. (Chengdu, China). 4-Dimethylaminopyridine and 4-toluene sulfonyl chloride were obtained from Braun Technology Co., Ltd. (Beijing, China). 3-Bromo-1-propanol was obtained from Xien Si Opod Technology Co., Ltd. (Tianjin, China). The herb section of *Platycodon grandiﬂorus* (PGS) was obtained from the Zhangzhou Chinese Herbal Medicine Trading Center (Anhui, China). Kryptofix2.2.2 (K2.2.2) was purchased from Merck Corporation (Darmstadt, Germany). H_2_^18^O was obtained from Dibo Chemical Technology Co., Ltd (Shanghai, China). All other reagents and chemicals were of analytical purity and obtained from Concord Technology (Tianjin, China).

### Probe Synthesis

PH (100 mg, 0.269 mmol, 1 eq) was dissolved in acetone/N,N-dimethylformamide (4 mL/2 mL), K_2_CO_3_ (100 mg, 0.724 mmol, 2.5 eq) added, and then the mixture was stirred while heating for 30 min at 60°C. 3-Bromo-1-propanol (67 μL, 3 eq) was dissolved in 1 mL of acetone and added dropwise to the system; then, the reaction was performed at 60°C for 12 h (Muthyala et al., 2014). The reaction was monitored by thin layer chromatography. The mixture was extracted by ethyl acetate (3 × 10 mL). The organic phase was washed repeatedly with saturated brine. The combined organic solvent was dried with Na_2_SO_4_ and concentrated *in vacuo*. The crude product was purified by column chromatography (petroleum ether:ethyl acetate = 2:1) to get a white solid, H-PH (75 mg, 65%). The NMR and high-resolution mass spectrometry (HRMS) of H-PH were ^1^H NMR (400 MHz, CDCl_3_) δ 6.95–6.91 (m, 2H), 6.91–6.83 (m, 4H), 4.88 (dd, J = 5.6, 1.7 Hz, 1H), 4.45 (d, J = 7.1 Hz, 1H), 4.19 (td, J = 5.8, 2.9 Hz, 2H), 4.14 (dd, J = 9.4, 1.2 Hz, 1H), 3.92–3.83 (m, 13H), 3.33 (dtd, J-12.4, 8.4, 8.0, 5.0 Hz, 2H), 2.96–2.89 (m, 1H), 2.32 (s, 1H), 2.08 (dt, J = 6.2, 4.7 Hz, 2H). HRMS [M + Na]^+^ calculated 453.1884, found 453.1886.

H-PH (60 mg, 0.140 mmol, 1 eq) was added into 1.5 mL of anhydrous dichloromethane, and 4-dimethylaminopyridine (0.1–0.2 eq, catalytic amount), 4-toluene sulfonyl chloride (37 mg, 0.195 mmol, 1.2 eq), and triethylamine (56 μL, 2 eq) were added successively into the system. Then the mixture was stirred at 25°C for 12 h (Lankshear et al., 2008). The reaction was monitored by thin layer chromatography. The mixture was extracted by ethyl acetate (3 × 10 mL). The organic phase was washed repeatedly with saturated brine. The combined organic solvent was dried with Na_2_SO_4_ and concentrated *in vacuo*. The crude product was purified by column chromatography (petroleum ether:ethyl acetate = 4:1) to get a white solid, T-PH (59 mg, 75%). The NMR and HRMS of T-PH were ^1^H NMR (400 MHz, CDCl_3_) δ 7.76 (dd, J = 8.3, 3.9 Hz, 2H), 7.29–7.25 (m, 2H), 6.95–6.75 (m, 6H), 4.87 (t, J = 5.4 Hz, 1H), 4.45 (dd, J = 7.2, 2.1 Hz, 1H), 4.27 (td, J = 6.0, 4.2 Hz, 2H), 4.14 (d, J = 9.4 Hz, 1H), 4.00 (td, J = 6.0, 4.0 Hz, 2H), 3.92–3.81 (m, 11H), 3.40–3.28 (m, 2H), 2.92 (ddd, J = 14.2, 7.5, 4.2 Hz, 1H), 2.40 (d, J = 4.5 Hz, 3H), 2.15 (td, J = 6.0, 4.4 Hz, 2H). HRMS [M + Na]^+^ calculated 607.1972, found 607.1978.

A cyclotron-accelerated proton bombardment of oxygen-enriched water was performed to obtain an oxygen-rich aqueous solution rich in [^18^F] fluoride ions, and [^18^F] fluoride ions were adsorbed on the column through an anion exchange column (Sep-Pak Light QMA cartridge, Waters, USA). The [^18^F] fluoride ion was eluted from the QMA column into the reaction tube with a solution of K_2_CO_3_, and Kryptofix 2.2.2 (2 mL) and the reaction tube was heated to 105°C to dry the reaction tube completely. Then, anhydrous acetonitrile (2 mL) was added, and the reaction tube was dried again at 105°C. The precursor compound (T-PH) in anhydrous DMSO (1 mL, 3 mg/mL) was added to the reaction tube and reacted at 110°C for 20 min; then, the reaction solution was diluted with 40 mL of deionized water and passed through a solid phase (Constantinescu et al., 2013; Jiang et al., 2018). The labeled products were adsorbed by the SepPak plus C-18 column (C-18), and the C-18 column was washed thrice with 10 mL water and the product eluted into a clean vial with 500 μL of absolute ethanol (57%). Three samples containing 400 μL of phosphate buffered saline (PBS) (pH 7.4) were tested for stability of the markers, and three parts of 100 μL of the labeled product were separately added to it and incubated at 37°C from 1 to 4 h. A small amount of sample was taken at each time point for dilution, and the stability of the labeled product in PBS was examined by radioactive ultra-high-pressure liquid chromatography (UPLC). Production of the desired [^18^F]-PH product was repeated with consistent quality on a Modular-Lab synthesizer.

### Preparation of Total Saponins

PGS (1 kg) was added to 5 L of pure water, soaked overnight, heated, boiled for 1 h, and filtered to obtain a filtrate. This process was repeated thrice, the filtrate combined, and the platycodon extract was obtained by rotary evaporation (Ahn et al., 2018). The extract of PG was dissolved in 70% ethanol overnight, and the upper layer was concentrated, re-dissolved in water, and purified on a macroporous adsorption resin to obtain total saponins of PG (PG-TS, 12 g, 1.2%) (Zhou et al., 2017).

### UPLC/Q-TOF-MS Analysis

The Waters UPLC system was used to analyze the samples (Waters Co., Milford, Massachusetts, USA). The sample was separated using an Acquity BEH C_18_ column (2.1 mm × 100 mm, Waters Co., Milford, Massachusetts, USA). The temperature was kept at 30°C, the flow rate was 0.4 mL/min, and the column equilibration lasted 5 min. The optimum mobile phase comprisedthe linear gradient system (A) 0.1% aqueous solution of formic acid and (B) acetonitrile: 0–5.0 min, B 5–20%; 5.0–20.0 min, B 20–30% and 20.0–30.0 min, B 30–100%. The injection volume was 2 μL. After the column separation, the 5% fraction was delivered directly to the quadrupole time-of-flight mass spectrometry (Q-TOF-MS). The detector was a dual electrospray ionization (ESI) probe. HRMS was obtained from Waters (Waters Corporation, Milford, USA), which was set in positive (ESI^+^) and negative (ESI^−^) ionization modes, and optimal analytical conditions were set as follows: the source temperature was 120°C, desolvated gas temperature flow was 350°C and 750 L/h, negative mode voltage was 3.0 kV, and positive voltage was 2.5 kV (Lei et al., 2018). The Q-TOF collection rate was 0.1 s, the delay was 0.02 s, and the first acquisition rate was 0.1 s. The resolution quadrupole (50–1,800 Da) was performed in wide-pass mode. Waters MassLynx^™^ (Waters Corp., Milford, MA, USA) was used for data collection and subsequent data processing.

### Preparation of Secondary Saponin

PG-TS (5 g) was hydrolyzed under basic conditions (pH 13, 100°C) for 3 h to obtain about 2.7 g of total secondary saponins. A part of the sugar ring was removed. GPD^682^ (128.5 mg, 4.76%) and 3-*O*-β-D-glucopyranosyl platyconic acid (GPA^696^) (15.9 mg, 0.59%) were obtained by flash chromatography (Biotage, Sweden), with ternary gradient elution (CH_2_Cl_2_:MeOH:H_2_O). GPD^682^ was obtained at CH_2_Cl_2_:MeOH:H_2_O = 8:2:0.2, and GPA^696^ was obtained at CH_2_Cl_2_:MeOH:H_2_O = 9.5:3:0.4 (Tang et al., 2017). The NMR and HRMS were described in the supporting information (**Figures S1−S8**).

### Analysis of Fluorescence Distribution in Cells

Human normal lung epithelial cells (BEAS-2B) were purchased from Shanghai Bogu Biotechnology Co., Ltd. (Shanghai, China) and cultured in RPMI-1640 medium (without Hepes) containing 10% fetal bovine serum and 1% diabody (100 U/mL). Cell culture conditions were as follows: temperature 37°C, CO_2_ 5%. The cells were cultured in confocal dishes and tested when the cells grew 80% to 90%. The cells were washed twice with PBS, then fluorescein isothiocyanate (FITC) (1 × 10^−5^ mmol) was added. The tests were divided into four groups according to the administration conditions: the control group was the normal culture, and the PG-TS (5 × 10^−4^ mg/mL), GPD682 (5 × 10^−4^ mg/mL), and GPA696 (5 × 10^−4^ mg/mL) dose groups were treated with corresponding platycodins. After incubation for 2 min, the changes in fluorescence distribution of FITC was observed under confocal microscopy (Leica, German). The excitation and emission wavelengths employed were 488 and 519 nm, respectively.

### Micro-PET Scanning

The animals used in this experiment were male Kunming mice (body weight 20 ± 2 g). The mice were randomly divided into the control and treatment groups. In the oral administration group, total saponins, PG-TS (50 mg/kg) were orally administered 1 h before the experiment, and the same amount of normal saline was administered to the control group. In the injection group, GPD^682^ (2.5 mg/kg) was intraperitoneally injected 30 min before the experiment, and the same amount of normal saline was administered as a control. All groups of mice were anesthetized with 2% isoflurane and simultaneously injected a certain amount of labeled [^18^F]-PH *via* tail vein, and all mice were immediately placed on the scanning surface and sent into the mouse imaging chamber. Then, a 120 min dynamic Micro-PET scan was performed using an Inveon-specific PET scanner (Siemens Inveon, Munich Germany). The average delay between the injection and the start of the PET scan was approximately 3 min. Mice remained stable under 2% isoflurane anesthesia throughout the scan. All motion pictures were automatically corrected for the effects of radioactive decay.

### Image Processing

The images were collected by IAW (Inveon acquisition workplace). After the data were obtained, the image was reconstructed with OSEM3D and displayed in 6 × 1, 7 × 2, 8 × 5, and 6 × 10 min. ASI Pro VM^™^ was used to map 3D volumes on organs (liver, brain, lung, heart, and kidney). Each volume of interest area was smaller than the actual size of the organ to reduce the interference of adjacent organ spillover effects on the experimental results. The data were corrected by attenuation and calculated to obtain ID %/g values at different time points for each organ.

### Statistical Analysis

All data were expressed as mean ± SD; statistical comparisons between groups were performed using the Student’s *t*-test. *P* value < 0.05 was considered a statistically significant difference.

## Results and Discussion

### Synthesis of [^18^F]-PH Probe

**Figure 1:**
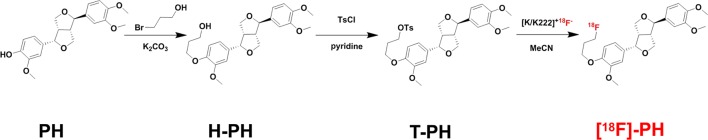
Synthesis strategy of [^18^F]-PH.

The [^18^F] modified PH probe was synthesized and used to evaluate the capacity of PG-TS and SSM for changing the distribution or delivery of the drug *in vivo*. The synthesis route is shown in **Figure 1**. The structure and purity of the synthesized product were identified by LC-MS and NMR.

### PG-TS Affects Probe Biodistribution

**Figure 2:**
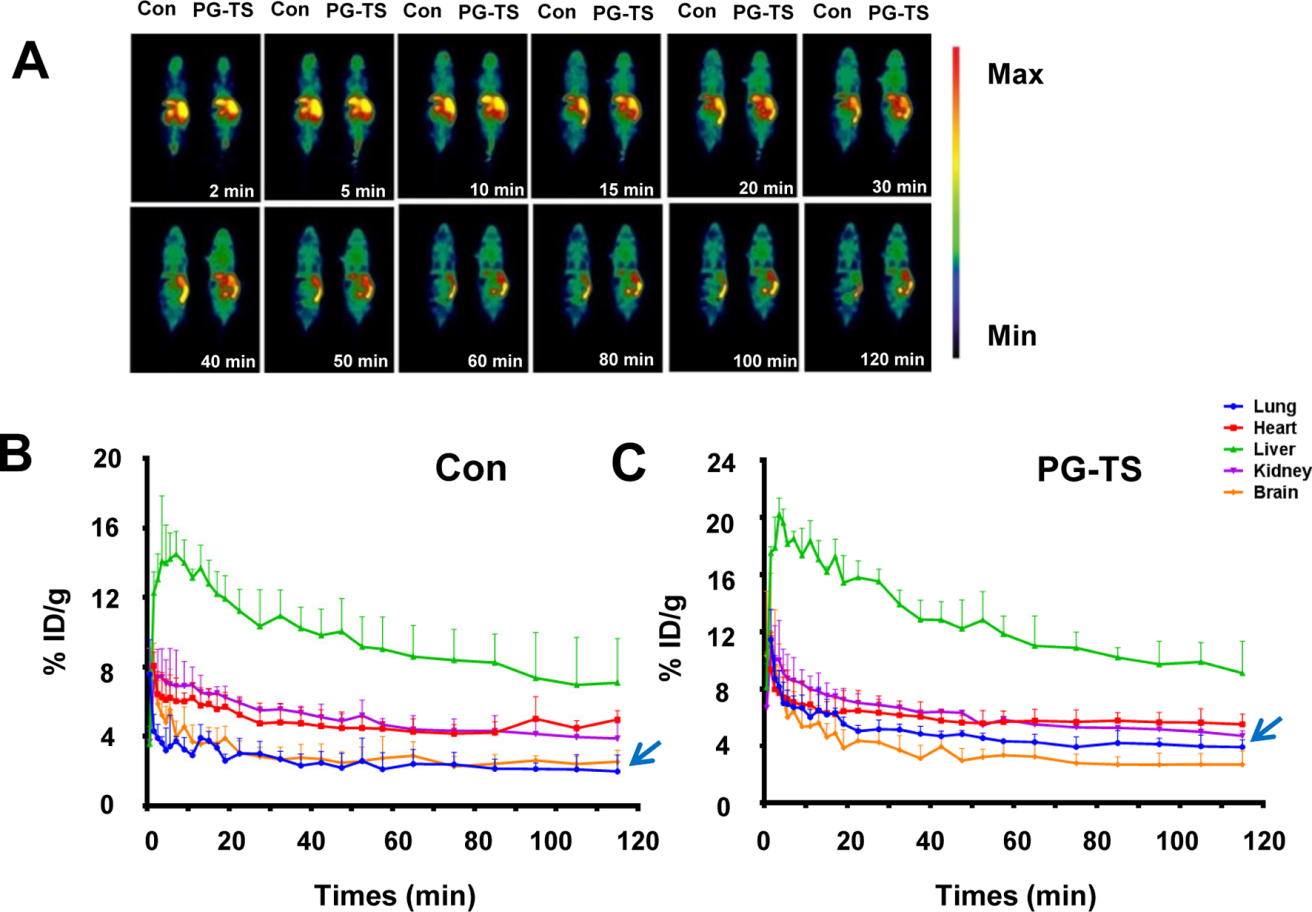
Biodistribution of [^18^F]-PH after oral administration of PG-TS. **(A)** Whole-body biodistribution of [^18^F]-PH over time. **(B)** The control group (only injected with [^18^F]-PH) of different organs uptake over time (expressed as a percentage of the injected dose). The vertical bars represent the standard deviation (*n* = 3). **(C)** Average uptake data of the mice after oral PG-TS intervention (expressed as a percentage of the injected dose). The vertical bars represent the standard deviation (*n* = 3).

The biodistribution image of the radioactive imaging agent was detected in the first 120 min after injection of the [^18^F]-PH probe, and results are presented in **Figure 2A**. The effect of PG-TS on the intake of [^18^F]-PH was varied in different organs. The brain, lungs, and heart reached the maximum intake immediately, and then decreased with time; the liver reached a peak in 5 min and then began to decline. **Figure 2B** shows the control group with only [^18^F]-PH probe; the average uptake data of the mice after oral PG-TS intervention is shown in **Figure 2C**. The physiological state of the mice was normal during the Micro-PET scan. As shown in **Figures 2B, C**, administration with PG-TS could significantly improve the dosimetry of the probe in the lung tissue (blue arrow). The result is consistent with previous studies (Cui et al., 2018). Herein, the [^18^F]-PH probe displayed good performance and visually showed the distribution of the progenitor *in vivo* before and after the treatment of PG-TS.

### GPD^682^ and GPA^696^ Constitute the Main SSM From Platycodins

**Figure 3:**
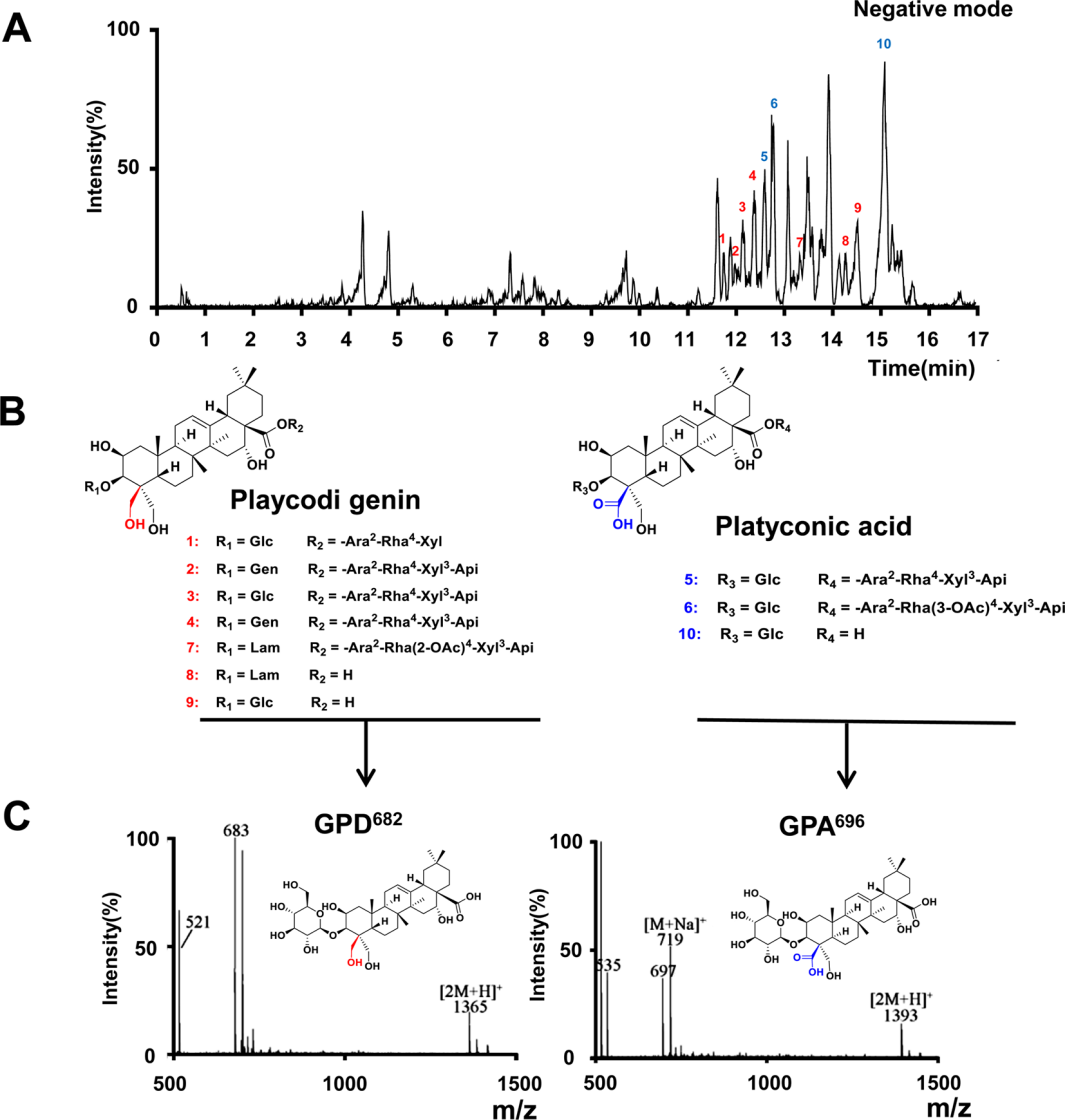
Platycodins of GPD^682^ and GPA^696^ were the main SSM. **(A)** Typical base peak intensity (BPI) chromatogram of PG-TS obtained in the ESI negative mode. **(B)** Classification of saponins in PG-TS. **(C)** Identification of GPD^682^ and GPA^696^ by Q-TOF-MS (in positive mode).

To screen the active ingredients in PG-TS, the optimal UPLC/Q-TOF-MS conditions were employed for the analysis of PG-TS. Molecular ion information was obtained in negative ion mode. Tenplatycodins that belong to platycodigenin and platyconic acid were identified by MS data (**Figure 3A**). The detailed retention time and MS data are shown in **Table 1**. The structures of seven platycodigenins and three platyconic acid derivatives are shown in **Figure 3B**. Although the number of secondary saponins may be large when orally administered with PG-TS, we found the main metabolites were GPD^682^ and GPA^696^, which have been detected in mice blood by LC-MS (in positive ion mode) in our previous study (Tang et al., 2017) (**Figure 3C**). Thus, we speculated that GPD^682^ and GPA^696^ might be the key SSM which further exert pharmacodynamic activity for changing the biodistribution of the drug to the lung tissue.

**Table 1:** UPLC-Q/TOF-MS identification of the platycosides in PG-TS.

No.	t_R_/min	Identification	Formula	MW	m/z of [M-H]-
1	11.75	deapioplatycodin D	C_52_H_84_O_24_	1092.5353	1091.5286
2	12.04	platycodin D3	C_63_H_102_O_33_	1386.6303	1385.6052
3	12.14	platycodin D	C_57_H_92_O_28_	1224.5775	1223.5605
4	12.37	platycodin A	C_59_H_94_O_29_	1266.5881	1265.5720
5	12.59	platyconic acid A	C_57_H_90_O_29_	1238.5568	1237.5431
6	12.74	platyconic acid B	C_59_H_92_O_30_	1280.5673	1279.5504
7	13.31	2″-O-acetylplatycodin D2	C_65_H_104_O_34_	1428.6409	1427.6208
8	14.26	platycoside K	C_42_H_68_O_17_	844.4457	843.4336
9	14.52	GPD^682^	C_36_H_58_O_12_	682.3928	681.3853
10	15.10	GPA^696^	C_36_H_56_O_13_	696.3615	695.3651

### Effect of Membrane Permeability by SSM *In Vitro*

**Figure 4:**
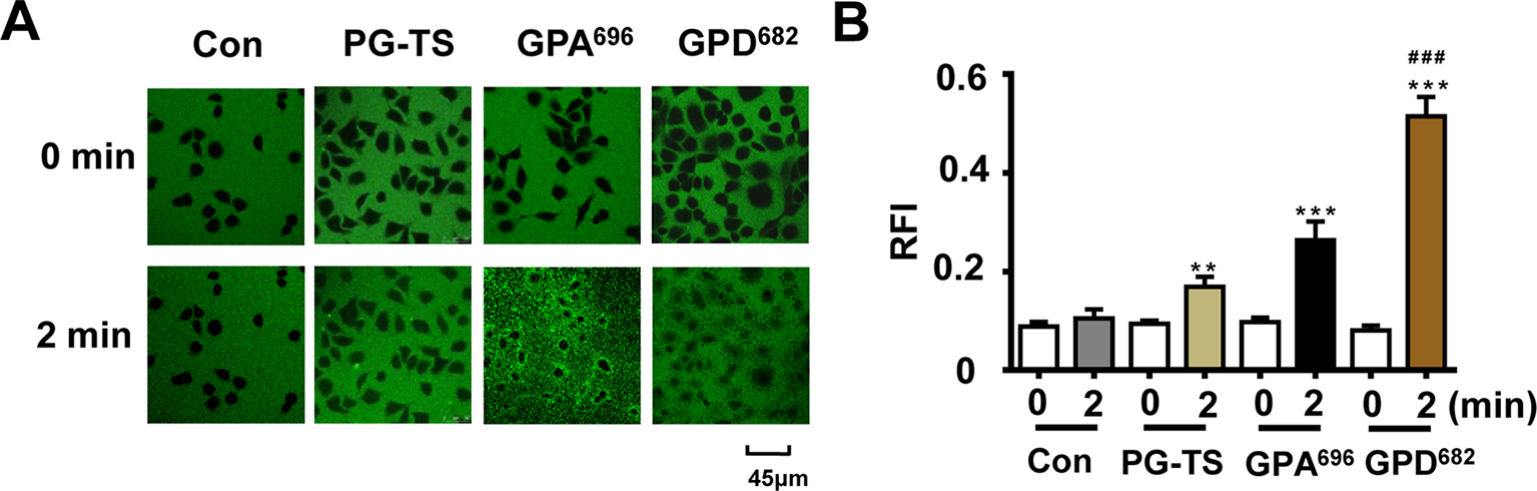
Effect of membrane permeability. **(A)** Control, PG-TS, GPA^696,^ and GPD^682^ group comparison of fluorescent dyes entering BEAS-2B cells at 0 and 2 min. **(B)** Ratio of relative fluorescence intensity (RFI) inside and outside the cell. Each bar represents the mean ± SD. **p < 0.01 *vs*. control; ***p < 0.001 *vs*. control; ^###^ *p* < 0.001 *vs*. GPA^696^ (*n* = 10).

To verify our hypothesis, the two SSM, GPD^682^ and GPA^696^, were purified and used to evaluate their membrane permeability at the cellular level. During the dynamic observation, it was found that the FITC did not enter the BEAS-2B cell rapidly in the control group and the PG-TS group. In the GPA^696^ group, the FITC was enriched around the cell membrane but was not seen entering the cell distinctly. However, GPD^682^ could promote FITC entry into the cell obviously within 2 min (**Figure 4A**). To show this difference better, ImageJ (https://imagej.nih.gov/) was used to randomly select 10 sets of intracellular and extracellular relative fluorescence intensity areas in adjacent regions, and the histogram was plotted with GraphPad Prism 6 (**Figure 4B**). After the intervention, the relative fluorescence intensity of cells in the GPD^682^ group could reach twice that of the GPA^696^ group and about thrice that of the PG-TS group. The advantage indicated that GPD^682^ might be the core SSM of PG-TS.

### GPD^682^ Improves Drug Delivery to Lung Tissue

**Figure 5:**
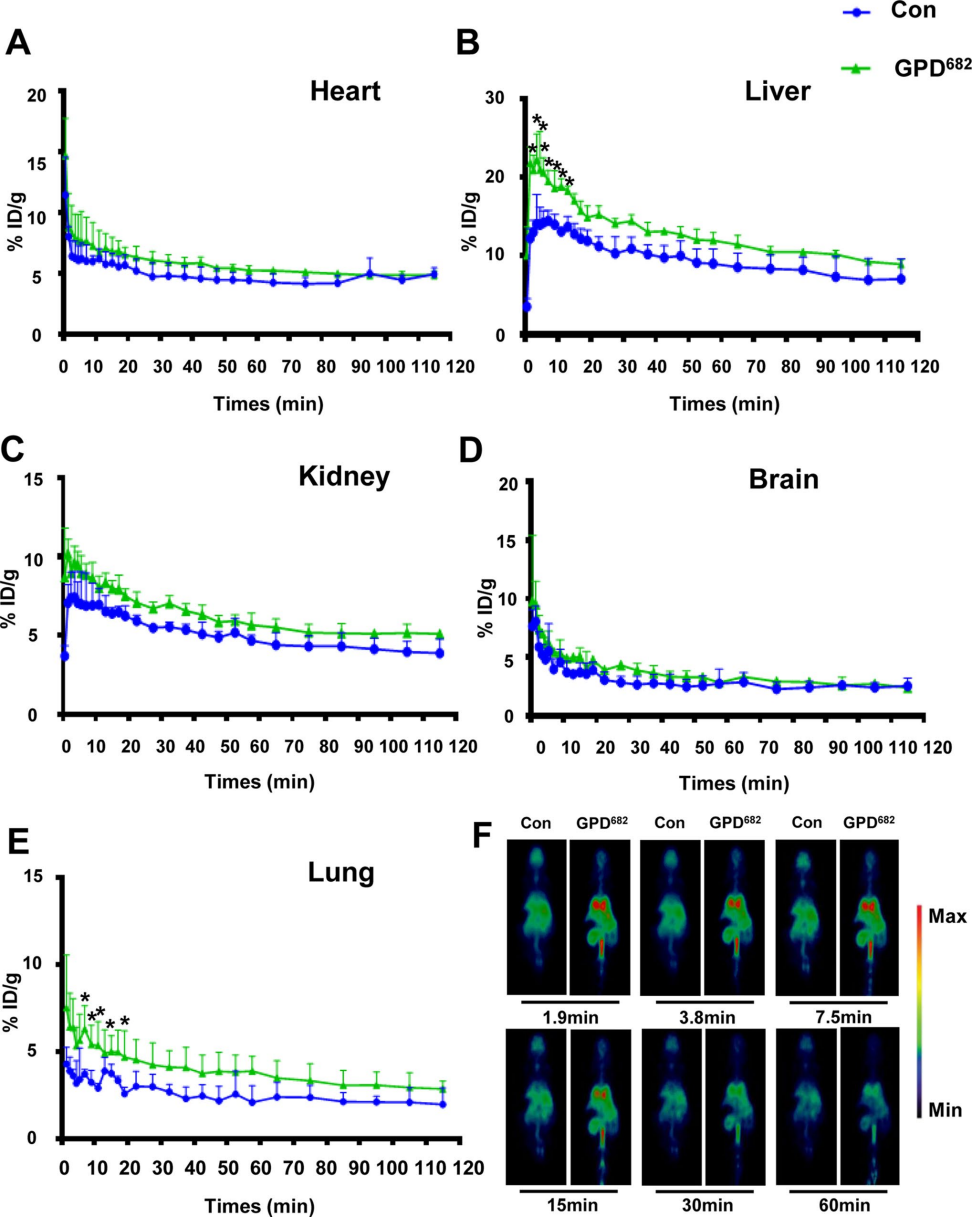
GPD^682^ improves drug delivery and distribution. **(A–E)** Blood concentration curve of each organ before and after GPD^682^ intervention. Each bar represents the mean ± SD. *p < 0.05 *vs*. control (*n* = 3). **(F)** [^18^F]-PH biodistribution in the lung (control *vs*. GPD^682^ influence).

Owing to the differences in the membrane permeability experiments, another Micro-PET test was designed to verify the effect of GPD^682^ *in vivo*. The change in the pharmacokinetic curve of [^18^F]-PH in mice with or without GPD^682^ intervention was plotted by using GraphPad Prism 6. As shown in **Figures 5A–E**, there was no significant change in the kidney, heart, and brain after GPD^682^ was injected for the intervention compared to the control group. However, significant changes were observed in the liver and especially in the lungs. For visual comparative analysis, the results of radiographic imaging of whole animals at 1.9, 3.8, 7.5, 15, 30, and 60 min are shown in **Figure 5F**. The data intuitively indicated that the lungs showed significant differences before and after GPD^682^ intervention. Compared to the control group in the first 15 min, the [^18^F]-PH concentration in the lungs could reach about 1.6 times, and the difference was not significant after. The activity of GPD^682^ may be attributed to its specific amphiphilic saponin molecule, which changed the membrane permeability (Lorent et al., 2014).

## Conclusion

In this study, the mechanism of prescription compatibility for *P. grandiflorum* was investigated. A key SSM, GPD^682^, was found in PG-TS, which effectively improves the distribution of drugs both *in vitro* and *in vivo*. The activity was confirmed by fluorescence imaging and Micro-PET scanning techniques. GPD^682^ may be utilized to improve the drug delivery targeted to the lung tissue as the potential assisting agent and further improve the therapeutic effect on respiratory disease.

## Data Availability

The raw data supporting the conclusions of this manuscript will be made available by the authors, without undue reservation, to any qualified researcher.

## Ethics Statement

This study was carried out following the recommendations of the Principle of Laboratory Animal Care (NIH Publication No. 85-23, revised 1985) guidelines, Animal Ethics Committee of the Nankai University. The protocol was approved by the Animal Ethics Committee of the Nankai University.

## Author Contributions

GB and YH designed the study. FS performed the experiments, analyzed the data, and drafted the manuscript. WW performed extraction and separation operations. MZ performed cell experiments. XM, QC, ZT, HH, TT, and LY assisted with experiments. YH and ZJ contributed to data discussion and reviewed the manuscript. GB reviewed the final manuscript, and all the authors have read and approved the final version.

## Funding

This research was supported by International Cooperation and Exchange of the National Natural Science Foundation of China (No. 81761168039)-Macau Science and Technology Development Fund (No. 015/2017/AFJ), National Natural Science Foundation of China (No. 81673616), and the State Key Laboratory of Medicinal Chemical Biology (Nankai University) (No. 2018083).

## Conflict of Interest Statement

The authors declare that the research was conducted in the absence of any commercial or financial relationships that could be construed as a potential conflict of interest.
