# Supplementary material for: Micro-PET Imaging Demonstrates 3-O-β-D-Glucopyranosyl Platycodigenin as an Effective Metabolite Affects Permeability of Cell Membrane and Improves Dosimetry of [18F]-Phillygenin in Lung Tissue
Source: Front Pharmacol. 2019 Sep 13;10:1020. doi: 10.3389/fphar.2019.01020 (PMC6753856; doi:10.3389/fphar.2019.01020)
Supplement: Supplementary file 1 [file Figure_1.docx]

# Supplemental Information

**Micro-PET imaging demonstrates 3-O-β-D-glucopyranosyl platycodigenin as an effective metabolite affects permeability of cell membrane and improves dosimetry of [^18^F]-phillygenin in lung tissue**

**Fukui Shen^1^, Wenbo Wu^1^, Man Zhang^1^, Xiaoyao Ma^1^, Qingxin Cui^1^, Zhongyao Tang^1,2^, Hao Huang^2^, Tiantian Tong****^2^, Leefong Yau^2^, Zhihong Jiang^2^,** **Yuanyuan Hou^1^* and Gang Bai^1^***

^1^State Key Laboratory of Medicinal Chemical Biology, College of Pharmacy and Tianjin Key Laboratory of Molecular Drug Research, Nankai University, Haihe Education Park, 38 Tongyan Road, Tianjin 300353, People’s Republic of China.

^2^State Key Laboratory of Quality Research in Chinese Medicine, Macau University of Science and Technology, Avenida Wai Long, Taipa, Macau, People’s Republic of China

## 1. General Information

High-resolution mass spectra (HRMS) were obtained with a FTICR-MS (Ion spec 7.0T) spectrometer. ^1^H NMR spectra were obtained by using a Bruker AV 400. Chemical shifts are reported in parts per million (ppm) relative to either a tetramethylsilane internal standard or solvent signals. Data are reported as follows: chemical shift, multiplicity (s = singlet, d = doublet, t = triplet, q = quartet, br = broad, m = multiplet), coupling constants and integration. ^13^C NMR spectra were recorded using a Bruker AV 400 spectrometer (100 MHz) using DMSO-*d*6 as the solvent. Chemical shifts (*δ*) are reported in parts per million measured relative to the solvent peak.

**2. Spectrum Data**

**Figure S1.** Chemical structure of 3-*O*-β-D-glucopyranosyl platycodigenin (GPD^682^).

The NMR and HRMS of GPD^682^ were: ^1^H NMR (400 MHz, DMSO-*d*6) δ 5.48 (s, 1H), 5.22 (d, J = 3.9 Hz, 1H), 4.97 (s, 2H), 4.71 (d, J = 4.5 Hz, 1H), 4.53 (dt, J = 18.4, 5.1 Hz, 2H), 4.32 (s, 1H), 4.23 (d, J = 7.7 Hz, 1H), 4.11 (s, 1H), 4.05-3.96 (m, 3H), 3.80-3.61 (m, 3H), 3.49-3.36 (m, 3H), 3.15 (d, J = 13.3 Hz, 4H), 3.05 (d, J = 8.0 Hz, 2H), 2.88 (dd, J = 14.3, 4.5 Hz, 1H), 2.21 (t, J = 13.4 Hz, 1H), 1.84 (tdd, J = 28.1, 10.0, 5.9 Hz, 4H), 1.72-1.45 (m, 5H), 1.31 (s, 6H), 1.17 (d, J = 15.8 Hz, 5H), 1.09-1.03 (m, 1H), 0.96 (dd, J = 12.4, 4.4 Hz, 1H), 0.90 (s, 3H), 0.83 (s, 3H), 0.67 (s, 3H); ^13^C NMR (100 MHz, DMSO-*d*6) δ 178.2, 144.0, 121.5, 104.3, 76.9, 76.8, 73.8, 73.0, 70.2, 68.3, 61.6, 61.1, 48.6, 47.4, 46.7, 46.5, 46.4, 44.2, 41.2, 36.3, 35.2, 34.6, 32.9, 32.7, 31.5, 30.3, 26.5, 24.2, 23.1, 18.2, 17.2, 16.8. HRMS [M - H]^-^ calculated 681.3856, found 681.3853.

**Figure S2.** The ^1^H NMR data of GPD^682^.

**Figure S3.** The ^13^C NMR data of GPD^682^.

**Figure S4.** The heteronuclear single quantum coherence (HSQC) NMR data of GPD^682^.

**Figure S5.** Chemical structure of 3-*O*-β-D-glucopyranosyl platyconic acid (GPA^696^).

The NMR and HRMS of GPA^696^ were: ^1^H NMR (400 MHz, DMSO-*d*6) δ 5.21 (d, J = 3.7 Hz, 2H), 5.04 (s, 2H)4.76 (d, J = 18.1 Hz, 1H), 4.33 (s, 2H), 4.21 (s, 1H), 3.79 - 3.61 (m, 4H), 3.43 (dd, J = 11.4, 5.2 Hz, 5H), 3.16 (d, J = 7.8 Hz, 2H), 3.04 (pd, J = 8.9, 3.7 Hz, 3H), 2.88 (dd, J = 14.1, 4.5 Hz, 1H), 2.21 (t, J = 13.5 Hz, 1H), 1.81 (d, J = 32.0 Hz, 5H), 1.73-1.54 (m, 3H), 1.44 (dd, J = 23.7, 9.0 Hz, 3H), 1.31 (d, J = 6.8 Hz, 4H), 1.23 (s, 1H), 1.21-1.11 (m, 2H), 1.06 (d, J = 9.0 Hz, 4H), 0.90 (s, 4H), 0.83 (s, 3H), 0.67 (s, 3H); ^13^C NMR (100 MHz, DMSO-*d*6) δ 178.3, 144.2, 121.3, 104.4, 76.9, 76.8, 73.8, 72.9, 70.1, 68.3, 61.1, 55.1, 48.6, 47.7, 47.3, 46.4, 46.3, 41.2, 35.9, 35.2, 34.6, 32.9, 32.3, 31.5, 30.3, 26.5, 25.5, 24.2, 23.3, 16.9, 14.9. HRMS [M - H]^-^ calculated 695.3648, found 695.3651.

**Figure S6.** The ^1^H NMR data of GPA^696^.

**Figure S7.** The ^13^C NMR data of GPA^696^.

**Figure S8.** The heteronuclear single quantum coherence (HSQC) NMR data of GPA^696^.
